# Plastid evolution: gene transfer and the maintenance of 'stolen' organelles

**DOI:** 10.1186/1741-7007-8-73

**Published:** 2010-06-10

**Authors:** Eunsoo Kim, John M Archibald

**Affiliations:** 1 Canadian Institute for Advanced Research, Program in Integrated Microbial Biodiversity, Department of Biochemistry and Molecular Biology, Sir Charles Tupper Medical Building, Dalhousie University, Halifax, NS B3 H 1X5, Canada

## Abstract

Many heterotrophic organisms sequester plastids from prey algae and temporarily utilize their photosynthetic capacity. A recent article in *BMC Genomics *reveals that the dinoflagellate *Dinophysis acuminata *has acquired photosynthesis-related genes by horizontal gene transfer, which might explain its ability to retain 'stolen' plastids for extended periods of time.

See research article http://www.biomedcentral.com/1471-2164/11/366

## Commentary

The evolution of plastids - the light-gathering organelles of eukaryotic algae and plants - was a pivotal event in eukaryotic evolution. A number of eukaryotic lineages have acquired photosynthesis directly from cyanobacteria (that is, primary endosymbiosis) or indirectly via secondary or even tertiary endosymbiotic events involving eukaryotes in the role of both host and endosymbiont [1]. The dinoflagellates, a phylum of unicellular eukaryotes containing both photosynthetic and heterotrophic members, are the undisputed champions of plastid acquisition, having obtained plastids from phylogenetically diverse algal groups. Using the latest transcriptome sequencing technologies, Wisecaver and Hackett [2], in a paper recently published in *BMC Genomics*, provide fascinating insight into the genetics and cell biology of the dinoflagellate *Dinophysis acuminata*, an organism whose 'stolen' plastids appear to be serviced by nucleus-encoded proteins of diverse evolutionary origins.

## Acquired phototrophy in dinoflagellates

**Figure 1:**
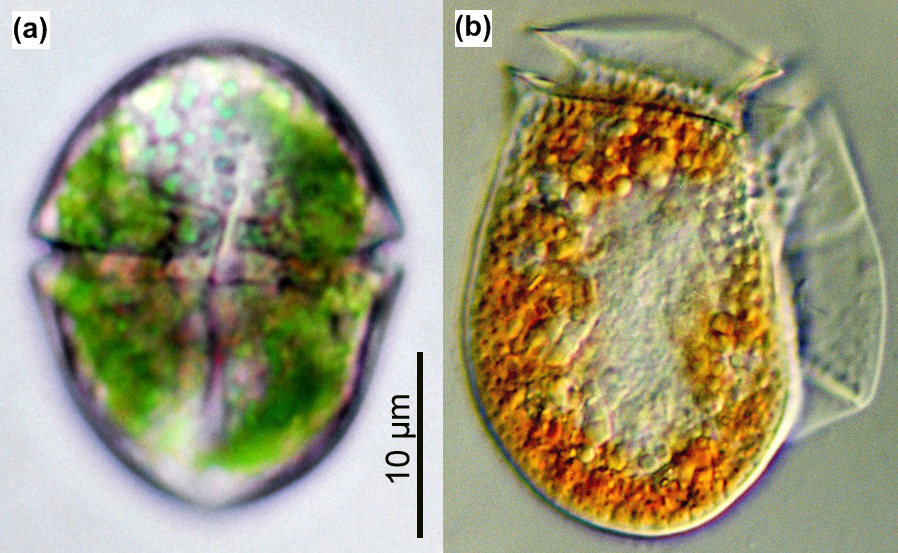
**Light micrographs of the dinoflagellates *Gymnodinium acidotum *and *Dinophysis fortii***. **(a) ***G. acidotum *harbors blue-green-colored, transient plastids that are derived from a *Chroomonas*-like cryptophyte alga. **(b) ***D. fortii *periodically captures cryptophyte-derived, orange-colored plastids from the 'photosynthetic' ciliate *Myrionecta rubra*. Scale information for (b) is not available. A normal cell of *D. fortii *is typically 51 to 83 μm long and 41 to 58 μm wide. (a) Photo courtesy of Lee W Wilcox and Gary J Wedemayer; (b) photo courtesy of Kazuhiko Koike and Kiyotaka Takishita.

Most photosynthetic dinoflagellates harbor canonical peridinin-pigmented plastids (of as yet unclear evolutionary origin), but a handful of species acquired their photosynthetic organelles from green algae, diatoms, and haptophytes [3]. Some heterotrophic dinoflagellates also perform 'acquired phototrophy' by harboring photosynthetic endosymbionts or sequestering plastids from prey, a phenomenon known as kleptoplastidy [4]. *Gymnodinium acidotum*, for example, acquires transient plastids from cryptophytes, a phylum of small unicellular algae (Figure 1a). In addition to the plastid, the dinoflagellate retains the 'nucleomorph' (the secondary endosymbiont nucleus), mitochondrion, and in some cases the nucleus of the engulfed cryptophyte [5]. In contrast, the dinoflagellate *Amphidinium wigrense *possesses three membrane-bound transient plastids of cryptophyte origin but does not retain a nucleomorph or any other endosymbiont-derived organelles [6].

By hosting the plastids of other organisms, dinoflagellates that are otherwise heterotrophic can supplement their diet by utilizing fixed carbon and other compounds provided by photosynthetic plastids [4]. Predictably, the extent to which dinoflagellate hosts depend on acquired phototrophy increases as the availability of prey decreases. When food is limiting, *D. acuminata *can obtain 45 to 100% of its entire carbon budget from its cryptophyte-derived, photosynthetic plastids, a fraction that decreases to only 10 to 30% when prey is abundant [7]. The retention time of transient plastids in dinoflagellates varies greatly depending on the species involved and the conditions under which they are grown (Table 1). For instance, cryptophyte-derived plastids of *Gymnodinium 'gracilentum' *persist for only 1 to 2 days whereas those of *Dinophysis caudata *remain active for around 2 months [8,9]. Collectively, these observations underscore the significance of mixotrophy (the combination of phototrophy and heterotrophy) for the survival and proliferation of dinoflagellates living in changing environmental conditions, but do little to shed light on how the stolen organelles maintain functionality for extended periods of time.

**Table 1:** Retention time of transient plastids in dinoflagellates under laboratory conditions

**Dinoflagellate host**	**Source of transient plastids**	**Retention time**	**Reference**
Dinophysis caudata	*Teleaulax *sp. (cryptophyte)^1^	Around 2 months	[8]
Dinophysis fortii	*Teleaulax amphioxeia* (cryptophyte)^1^	At least 40 days	[11]
Gymnodinium acidotum	*Chroomonas *sp. (cryptophyte)	At least 10 days	[5]
Gymnodinium 'gracilentum'	*Rhodomonas salina *(cryptophyte)	1-2 days	[9]
Pfiesteria piscicida	*Rhodomonas *sp. (cryptophyte)	At least 9 days^2^	[13]
Unnamed dinoflagellate	*Phaeocystis antarctica *(haptophyte)	5-8 months	[14]

^1^*D. caudata *and *D. fortii *cannot sequester plastids directly from the cryptophyte cells, and instead obtain them from the ciliate *Myrionecta rubra *that consumes cryptophytes.

^2^Retention time varies depending on light intensity.

## Plastids in *Dinophysis*

The dinoflagellate genus *Dinophysis *is broadly distributed in the ocean and currently includes over 100 species, some of which pose major economic and health concerns as agents of diarrhetic shellfish poisoning. *Dinophysis *is well known for its ability to sequester and utilize functional plastids of cryptophyte and, less commonly, haptophyte origins [4]. Curiously, strains of *Dinophysis *appear incapable of harvesting plastids directly from the algal cells; they acquire them indirectly by engulfing the ciliate *Myrionecta rubra*, a heterotroph that itself feeds on cryptophytes belonging to the *Teleaulax*/*Geminigera *clade [8]. In *M. rubra*, the cryptophyte-derived plastids exist as chloroplast (plastid)-mitochondrial complexes (CMCs), and one or more cryptophyte nuclei are retained separately from the CMCs in the ciliate host cytoplasm [4]. In contrast, cryptophyte-derived plastids of *Dinophysis *are bounded by two membranes and are devoid of nucleomorphs and mitochondria [8].

Whether or not the plastids of *Dinophysis *are permanent or transient has been the subject of much debate. Recent molecular data and culture experiments support the idea that *Dinophysis *plastids are most likely to be transient and need to be periodically 'replaced' by re-uptake of photosynthetic *M. rubra *[8,10]. However, differences in the ultrastructure of *Dinophysis *plastids and those of free-living cryptophyte cells suggest to some researchers that *Dinophysis *consumes *M. rubra *not to replace its plastids, but rather to obtain growth factors and other essential compounds [4].

In the case of the dinoflagellates *D. caudata *and *D. fortii*, the acquired cryptophyte plastids remain functional for well over a month in the absence of the cryptophyte nucleomorph and nucleus [8,11]. This is remarkable given that present-day plastids possess at most around 200 protein-coding genes, a fraction of the coding capacity of their cyanobacterial progenitors, and are dependent on the import of hundreds of nucleus-encoded proteins. Wisecaver and Hackett [2] surveyed the expressed gene set of *D. acuminata *in an effort to determine the extent to which its nuclear genome contains cryptophyte-derived genes for plastid-targeted proteins that could aid in the long-term stability of stolen plastids. In doing so, the authors took advantage of the 22-bp *trans*-spliced leader (SL) sequence present on mature dinoflagellate mRNAs, but not on those of ciliates or cryptophytes. *D. acuminata *poly(A)-primed cDNA was amplified using a 5' SL primer and sequenced using ultra-high-throughput 454 pyrosequencing. To control for the possibility of contamination, the transcriptomes of the ciliate *M. rubra *and the cryptophyte *Geminigera cryophila*, the organisms from which *D. acuminata *acquires its plastid, were also surveyed. The results are significant for two main reasons.

First, from a set of approximately 6,000 unique gene clusters, Wisecaver and Hackett [2] identified only five dinoflagellate nuclear genes that were strong candidates for being plastid-related - *psbU*, *petF*, and *psbM *and genes for the light-harvesting protein LI818 and the triose-phosphate transporter TPT. As predicted, the protein products of these five genes possess putative transit peptides: that is, amino-terminal leader sequences that are required to translocate host-synthesized proteins across the two inner plastid membranes in other algae [1]. *D. acuminata *thus appears to possess significantly fewer plastid-related genes in its nucleus than do dinoflagellates with permanent plastids, suggesting that transfer of additional genes for plastid-targeted proteins must occur if the stolen plastids are to be fully integrated into the host dinoflagellate. Second, sequence analyses reveal that only one of the plastid-related genes identified in *D. acuminata *(*psbM*) actually comes from the same source as its stolen plastid - that is, cryptophyte algae. The remaining four genes appear to have been acquired by horizontal gene transfer from other sources, including haptophytes and fucoxanthin-containing dinoflagellates [2].

The results of Wisecaver and Hackett [2] are particularly interesting when contrasted with the evolution of kleptoplastidy in the sea slug *Elysia chlorotica*, an organism that harvests plastids from the stramenopile alga *Vaucheria litorea *[12]. The transient plastids of *E. chlorotica *remain remarkably stable for up to 10 months in the absence of the algal nucleus. Recent studies have uncovered a number of genes for putative plastid-targeted proteins in the sea-slug nucleus, such as *psbO *(for photosystem II) and *fcp *(for light-harvesting complexes) [12]. These sea-slug genes have uniformly high sequence identities to homologs in *V. litorea*, suggesting that the genes were transferred relatively recently from the algal nucleus. This is in stark contrast to the situation in *D. acuminata*, whose putative plastid-targeted proteins have been acquired from multiple algal sources. This difference is perhaps explained by the trophic strategy of *D. acuminata*, which, in contrast to *E. chlorotica*, consumes a variety of prey, including many different photosynthetic algae, while performing acquired phototrophy. Exactly when *D. acuminata *acquired these 'foreign' genes is unclear. Some or all heterotrophic dinoflagellates are thought to be ancestrally photosynthetic [1], and so at least some of them might be holdovers from a time when *Dinophysis *species harbored more conventional plastids.

The advent of ultra-high-throughput sequencing has made it possible to obtain massive sequence datasets from experimentally challenging organisms - and even collections of intimately associated organisms - on a scale unimaginable even a few years ago. The results of Wisecaver and Hackett [2] represent a landmark in this regard, providing an important launch point for future dissection of the molecular and biochemical processes involved in dinoflagellate kleptoplastidy. Such experimentation will include definitive proof that the gene products in question are indeed targeted to the plastids in the context of *D. acuminata *cells, and even deeper transcriptome sequencing to further assess the degree of plastid proteome mosaicism in these intriguing organisms.
